# Atezolizumab Plus Chemotherapy vs. Chemotherapy in Advanced or Metastatic Triple-Negative Breast Cancer: A Cost-Effectiveness Analysis

**DOI:** 10.3389/fpubh.2021.756899

**Published:** 2021-10-29

**Authors:** Xiaoyan Liu, Yitian Lang, Yahui Liao, Yizhun Zhu

**Affiliations:** ^1^Department of Pharmacy, Huangpu Branch, Shanghai Ninth People's Hospital, Shanghai Jiao Tong University School of Medicine, Shanghai, China; ^2^State Key Laboratory of Quality Research in Chinese Medicine, School of Pharmacy, Macau University of Science and Technology, Taipa, Macau SAR, China

**Keywords:** atezolizumab, cost-effectiveness, partitioned survival model, triple-negative breast cancer, the perspective of China

## Abstract

**Purpose:** The IMpassion130 trial demonstrated the efficacy of adding atezolizumab to paclitaxel for advanced or metastatic triple-negative breast cancer (TNBC). The current study evaluated the cost-effectiveness of adding atezolizumab to nab-paclitaxel for TNBC from the perspective of Chinese health sector.

**Methods:** A partitioned survival model was implemented for patients with TNBC. The survival data were derived from IMpassion130 trial. Direct costs and utility values were collected from the Chinese Drug Bidding Database and published literatures. The primary analysis outcomes were quality-adjusted life-years (QALYs) and incremental cost-effectiveness ratios (ICERs). Sensitivity analyses were performed to observe model stability.

**Results:** In the base-case analysis, the ICER of atezolizumab plus nab-paclitaxel vs. nab-paclitaxel is respectively, $176,056/QALY, $118,146/QALY, and $323,077/QALY in the ITT, PD-L1(+) and PD-L1(–) group.

**Conclusion:** Adding atezolizumab to nab-paclitaxel could improve survival time significantly in the PD-L1-positive group, but it is not a cost-effective strategy compared to nab-paclitaxel monotherapy for Chinese patients with advanced or metastatic triple-negative breast cancer in the current economic context of China.

## Introduction

Breast cancer is the most common female malignant cancer and accounts for around one-tenth of all new diagnosed cancers worldwide (1). The incidence has been generally rising over the last 50 years with rapid increases observed particularly in developing countries (2, 3). Molecular markers depend primarily on the expression of relevant receptors in breast cancer, including estrogen receptor (ER), progesterone receptor (PR) and human epidermal growth factor receptor 2 (HER2). Various expressions of these receptors classified into major subtypes include luminal A, luminal B, HER2-overexpressing and triple-negative breast cancer (TNBC) (4–6). TNBC, an intrinsic subtype of breast cancer, is defined as a tumor that does not express the ER, PR, or HER2 and accounted for 15–20% of all breast cancer cases. As this type of cancer cell does not respond to hormone therapy or targeted drugs, it brings a big challenge to clinical treatment (7). Therefore, chemotherapy, especially chemotherapy with paclitaxel as the main component, is still the first choice for clinical therapeutic regimen (8, 9). However, there are still many new regimens being explored. In recent years, cancer immunotherapy has been applied to various cancers when no long-term responses were observed with cytotoxic chemotherapy. All immunotherapies have a similar mechanism of action that forces the body's own immune system to eliminate cancer cells. Programmed death-protein 1(PD-1)/programmed death ligand-1(PD-L1) pathway is one mechanism for tumor cells to avoid anti-tumor immune response. The reason is that when the PD-L1 of cancer cell binds to the PD-1 receptor of T cells, the expansion and activity of cytotoxic T cells are suppressed (10). However, the PD-1/PD-L1 inhibitors can block the pathway. At present, many clinical trials have been conducted for TNBC patients using PD-1/PD-L1 inhibitors (11). KEYNOTE-355 trial show that the progression-free survival (PFS) was 9.7 months with pembrolizumab plus chemotherapy and 5.6 months with placebo plus chemotherapy in CPS of 10 or more TNBC patients at the second interim analysis (12). IMpassion130 trial similarly explored the efficacy of immune checkpoint inhibitors (ICIs) adding to first-line treatment. Among TNBC patients with PD-L1(+), median progression-free survival was 7.5 months with atezolizumab plus chemotherapy, as compared with 5.0 months with placebo plus chemotherapy (13). And the overall survival was 25.0 months with atezolizumab plus chemotherapy and 18.0 months with placebo plus chemotherapy (14). It indicated that PD-1/PD-L1 inhibitors bring significant clinical benefits for PFS/OS in the PD-L1(+) TNBC patients. Therefore, some PD-1/PD-L1 inhibitors such as atezolizumab have been approved by FDA in 2019 (11). At present, atezolizumab is available in China, its price and economic burden are uncertain for Chinese patients. As far as we know, some studies have assessed the cost-effectiveness of atezolizumab for TNBC from the US payer perspective. However, no research has been conducted to analyze the potential economic burden of the therapeutic regimen from the Chinese perspective. We evaluated the cost-effectiveness of atezolizumab plus nab-paclitaxel vs. placebo plus paclitaxel in TNBC patients from the perspective of Chinese health sector.

## Materials and Methods

### Model Structure

In this study, we used a partitioned survival model to simulate the disease survival states of TNBC patients beyond the follow-up time of clinical trial and make a cost-effectiveness analysis. The target population of the study was kept with that of the IMpassion130 trial, who were aged 18 years or older and had been confirmed histologically documented, unresectable, locally advanced or metastatic TNBC. The patients of this study receive one of two interventions until disease progression: (1) chemotherapy (nab-paclitaxel); (2) atezolizumab plus chemotherapy. When suffered disease progressed, it is assumed that initial regimen became invalid and the current therapy regimen was terminated. And then, 53.7% of patients in the atezolizumab–nab-paclitaxel group and 60.3% in the placebo–nab-paclitaxel group receive subsequent best supportive anticancer regimens.

**Figure 1:**
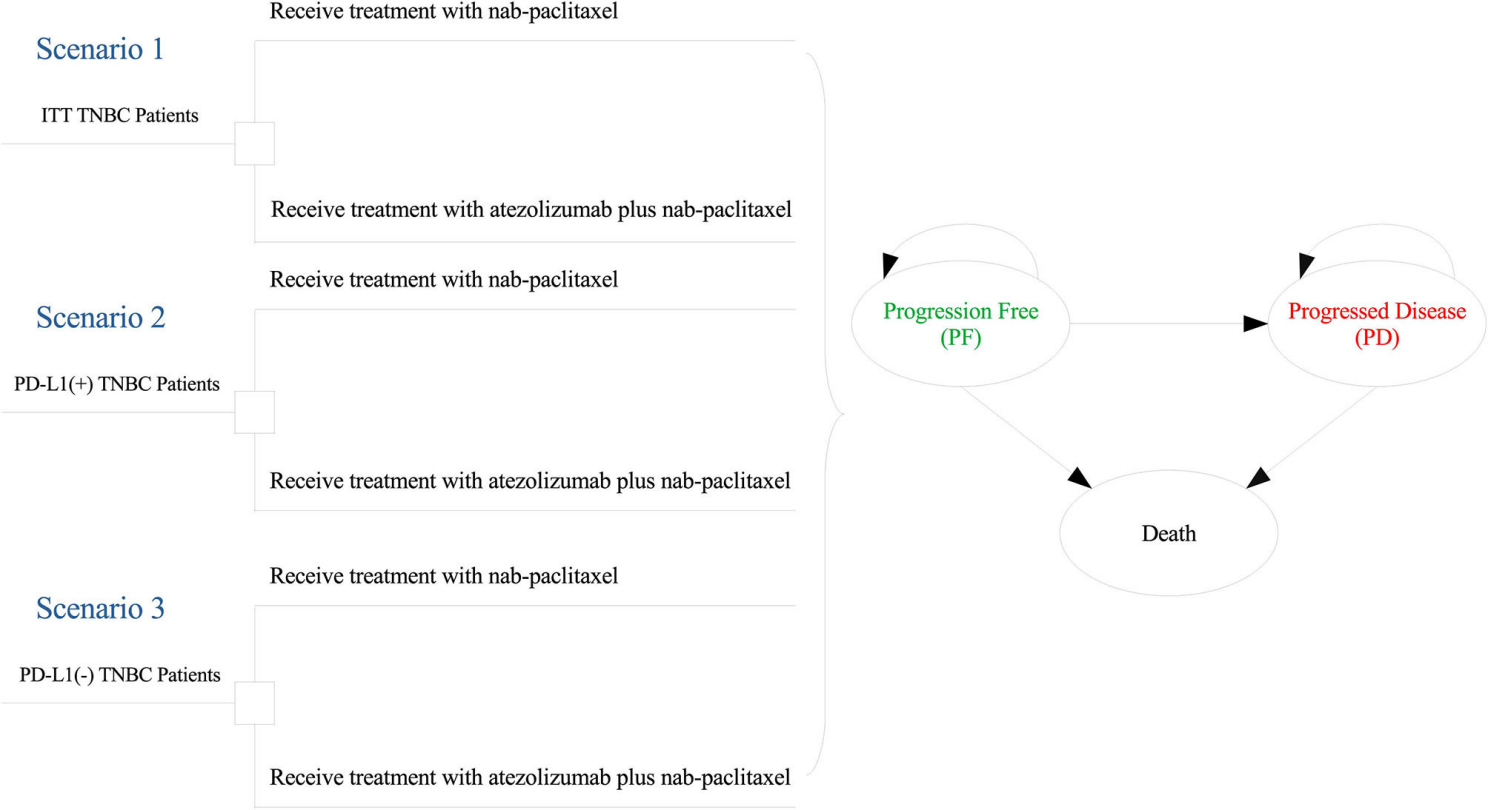
Model structure overview. ITT, intention-to-treat; PD-L1, programmed death ligand-1; TNBC, triple-negative breast cancer.

The partitioned survival model was composed of three mutually exclusive health states, which are respectively progression-free (PF) survival, progressed disease (PD) and death. Figure 1 shows the tree diagram and bubble diagram. The TNBC patients entered the model in PFS state primarily and then could move to the PD or death states based on survival data. The duration of the model cycle was 28 days, which was consistent with the treatment protocol in IMpassion130. Extrapolating short-term and limited results to reflect long-term prognosis can fully understand the outcome of the disease. So, the time horizon of 10 years was essential to ensure that TNBC patients fully entered the terminal state.

### Clinical Data

The available observational time of IMpassion130 trial was around 42 months for OS and PFS. To predict the survival outcomes over a 10-year horizon, a method that extrapolated over the follow-up time was used based on algorithms proposed by Guyot et al. (15). The tool of digitizing the OS and PFS Kaplan-Meier curves for interventions is Engauge Digitizer (version 12.1, https://github.com/markummitchell/engauge-digitizer/releases). The generated simulated individual patient level data (IPD) were applied to fit following parametric distributions: Weibull, Gompertz, exponential, log-normal, and log-logistic distributions. The best fitted distribution of IPD is selected based on Akaike information criterion (AIC) value (16).

### Costs and Utilities

This analysis was conducted from the perspective of the Chinese health sector. Only direct medical costs were considered, including costs related to drugs, management of adverse events and palliative care. The drug dose is consistent with that of the IMpassion130. In the chemotherapy regimen, nab-paclitaxel was used at a dose of 100 mg body surface area (BSA) per square meter on days 1, 8, and 15 of each model cycle. In atezolizumab plus chemotherapy regimen, atezolizumab was administered at a dose of 840 mg on days 1 and 15 of each model cycle and nab-paclitaxel is administered in keeping with the above-mentioned chemotherapy regimen. The mean BSA of Chinese patients is 1.72 m^2^ (17). The prices of nab-paclitaxel and atezolizumab in China were acquired from drug acquisition costs in local charge database (18). Costs related to terminal care and subsequent best supportive care (BSC) were derived from published literatures (19, 20). The IMpassion130 trial shared data about incidences of adverse events. And only the costs related to managing grade 3 and more AEs were included. Grade 1 and 2 AEs could be well-managed, the costs of management were not considered. The costs of managing grade 3–5 AEs were sourced from previously published economic studies (21, 22). All costs reported for years prior to 2020 were updated to 2020 US dollars (US$) using the health care services component of Chinese Consumer Price Index. More details about costs are summarized in Table 1.

**Table 1:** Input parameters of the model.

**Parameters**	**Expected value**	**Range**	**Distribution**	**References**
**Clinical inputs**				
**ITT TNBC patients**				
PFS: Atezolizumab + nab-paclitaxel	meanlog: 1.996 sdlog: 0.938	NA	Lognormal	(14)
OS: Atezolizumab + nab- paclitaxel	shape: 1.6892 scale: 22.1345	NA	Log-logistic	(14)
PFS: nab-paclitaxel	meanlog: 1.7856 sdlog: 0.941	NA	Lognormal	(14)
OS: nab-paclitaxel	shape: 1.3805 scale: 26.3036	NA	Weibull	(14)
**TNBC patients with PD-L1-positive status**				
PFS: Atezolizumab + nab-paclitaxel	meanlog: 2.0798 sdlog: 1.0586	NA	Lognormal	(14)
OS: Atezolizumab + nab- paclitaxel	meanlog: 3.2527 sdlog: 1.1196	NA	Lognormal	(14)
PFS: nab-paclitaxel	meanlog: 1.6705 sdlog: 0.975	NA	Lognormal	(14)
OS: nab-paclitaxel	shape: 1.301 scale: 25.56	NA	Weibull	(14)
**TNBC patients with PD-L1-negative status**				
PFS: Atezolizumab + nab-paclitaxel	shape: 1.934 scale: 6.657	NA	Log-logistic	(14)
OS: Atezolizumab + nab- paclitaxel	shape: 1.815 scale: 20.045	NA	Log-logistic	(14)
PFS: nab-paclitaxel	shape: 1.8035 scale: 6.2604	NA	Log-logistic	(14)
OS: nab-paclitaxel	shape: 1.456 scale: 26.4987	NA	Weibull	(14)
**Cost estimates**				
Atezolizumab (per 1,200 mg)	$4,756	3,567–5,945	Gamma	(18)
Nab-paclitaxel (per 100 mg)	$105.67	79.25–132.09	Gamma	(18)
Drug administration (per cycle)	$75.40	56.55–94.25	Gamma	(20)
Best supportive care (per cycle)	$1,886.67	1,415–2,358.34	Gamma	(19)
Terminal care (per patient)	$1,923.29	1,442.47–2,404.11	Gamma	(20)
**Costs of main adverse events**				
Fatigue	$131.78	98.84–164.73	Gamma	(22)
Anemia	$607.06	455.30–758.83	Gamma	(22)
Neutropenia	$526.90	395.18–658.63	Gamma	(22)
Neutrophil count decreased	$104.95	78.71–131.19	Gamma	(21)
**Utility estimates**				
Progression free survival	0.843	0.632–1	Beta	(23)
Progressed disease	0.60	0.45–0.75	Beta	(23)
**Disutility estimates**				
Utility reduction due to fatigue	−0.029	−0.022 to −0.036	Beta	(24)
Utility reduction due to anemia	−0.029	−0.022 to −0.036	Beta	(24)
Utility reduction due to neutropenia	−0.012	−0.050 to −0.083	Beta	(24)
**Probability of main grade 3-5 adverse events in atezolizumab plus nab-paclitaxel arm**				
Fatigue	3.8%	2.9–4.8%	Beta	(14)
Anemia	3.1%	2.3–3.9%	Beta	(14)
Neutropenia	8.4%	6.3–10.5%	Beta	(14)
Neutrophil count decreased	4.9%	3.7–6.1%	Beta	(14)
**Probability of main grade 3-5 adverse events in nab-paclitaxel arm**				
Fatigue	3.4%	2.6–4.3%	Beta	(14)
Anemia	3%	2.3–3.8%	Beta	(14)
Neutropenia	8.2%	6.2–10.3%	Beta	(14)
Neutrophil count decreased	3.7%	2.8–4.6%	Beta	(14)
**Other parameters**				
Body surface area	1.72	1.52–1.92	Normal	(17)

*All costs sourced from China in this study were converted into US dollars ($1 = RMB 6.8974 in 2020)*.

*BSC, best supportive care; ITT, intention-to-treat; PD-L1, programmed death ligand-1; TNBC, triple-negative breast cancer; PFS, Progression-free survival; OS, Overall survival*.

Every health state was assigned a health utility value in this partitioned survival model. Since the IMpassion130 trial lacked research about the quality-of-life data of TNBC patients, other robust data is extremely important. As the quality of life is associated with progressive stage, the utility values in metastatic/advanced breast cancer and TNBC were assumed to be consistent. The utility estimates for PF state and PD state were assumed to be 0.843 and 0.60 based on data collected from studies about locally advanced and metastatic breast cancer (23). In addition, the values of disutility due to grade 3–5 main AEs should be considered, the values were derived from relevant economic studies (24).

### Analyses

Our analyses covered three scenarios (Figure 1): intention-to-treat (ITT) TNBC patients (Scenario 1), PD-L1-positive (expression on tumor-infiltrating immune cells ≥ 1%) TNBC patients (Scenario 2) and PD-L1-negative (expression on tumor-infiltrating immune cells <1%) TNBC patients (Scenario 3). In each scenario, the treatment regimens received by the patients does not change and only the survival data of patients are different among all scenarios.

In the base-case analysis, we used incremental cost-effectiveness ratios (ICERs) to evaluate the incremental cost per additional quality-adjusted life-year (QALY) gained between atezolizumab plus nab-paclitaxel and nab-paclitaxel regimen. A 3% annual discount rate was applied for all costs and QALYs. If the ICER of atezolizumab plus nab-paclitaxel compared with nab-paclitaxel is below the willingness-to-pay (WTP) threshold, atezolizumab plus nab-paclitaxel regimen is considered “cost-effective.” The WTP threshold of three times the per capita gross domestic product (GDP) was recommended and is calculated to be $31,316 in China (25, 26).

In order to assess the robustness of our results and identify the variables that have considerable impacts on the analysis results, we conducted one-way and probabilistic sensitivity analyses (PSA) for model input parameters. In one-way sensitivity analyses, the range of every input parameter was assumed a variation by ±25%. The range of discount rate is between 0 and 8%. In addition, the PSA was conducted by a Monte Carlo simulation of 1,000 iterations. All input parameters were sampled simultaneously based on specific probability distributions. Health utilities, disutility values and probabilities of adverse events were sampled from Beta distribution, and the costs were sampled from Gamma distribution (27). The PSA outcomes are presented as cost-effectiveness acceptability curve (CEAC) to illustrate the likelihood that atezolizumab plus nab-paclitaxel regimen was cost-effective at specific WTP threshold. The partitioned survival model and cost-effectiveness analysis model were created and programmed in R (version 4.0.5, http://www.r-project.org).

## Results

### Validity of the Fitted Parametric Survival Function

**Figure 2:**
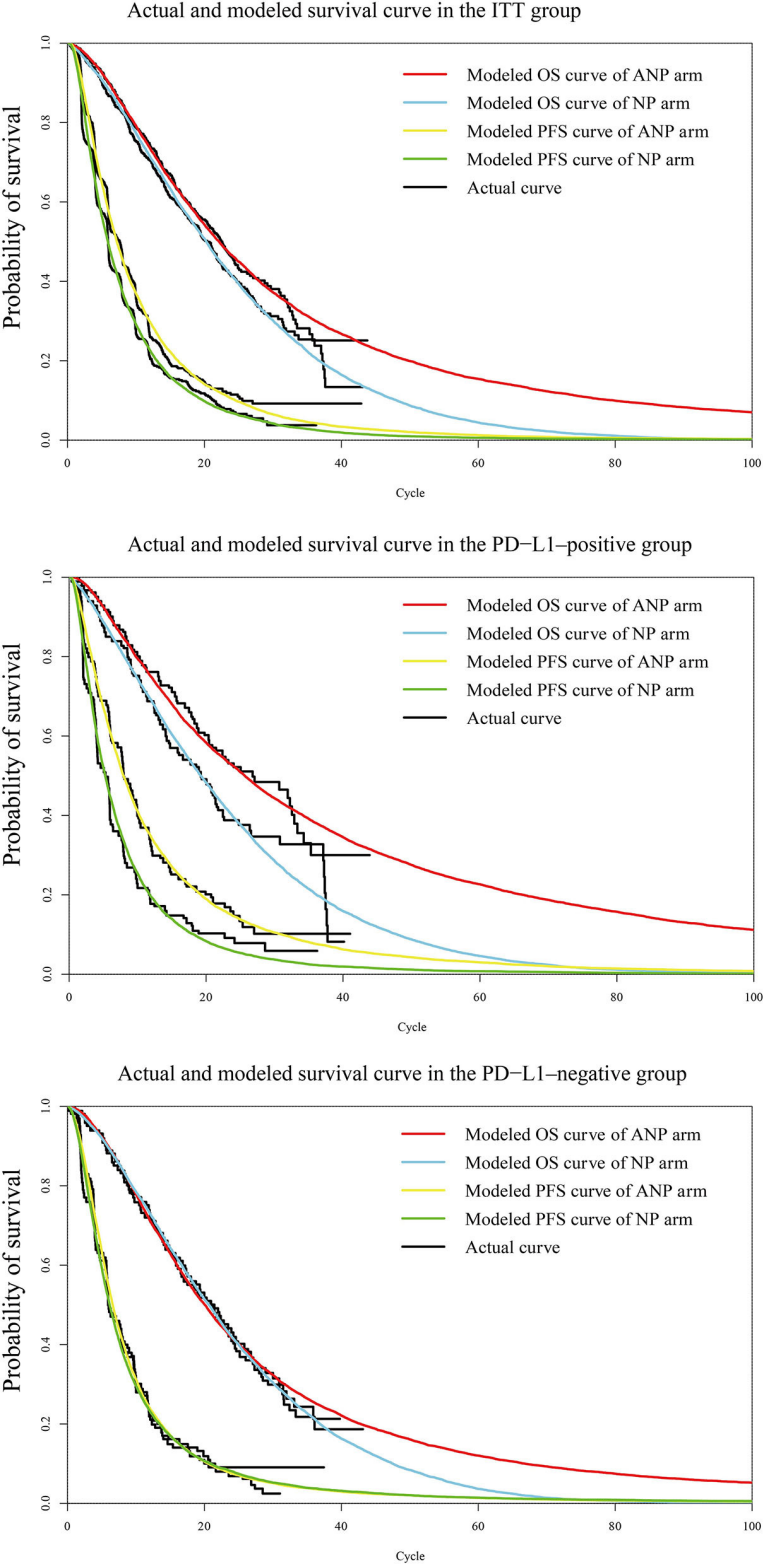
Diagram of modeled PFS and OS fit curves in different regimens. The colored lines represent the modeled survival curves, the black lines represent the actual survival curves. Each cycle of the x-axis is 4 weeks. PFS, progression-free survival; OS, overall survival; ITT, intention-to-treat; PD-L1, programmed death ligand-1. ANP, atezolizumab plus nab-paclitaxel; NP, nab-paclitaxel.

The validation by comparing the observational and predicted curves is shown in Figure 2. The distribution of projected curve could be seen in Table 1.

### Base-Case Analysis

All base-case results were summarized in Table 2.

**Table 2:** Results of the base-case analysis.

**Scenario**	**Regimen**	**Total cost (USD)**	**LYs**	**QALYs**	**CER (USD/LY)**	**CER (USD/QALY)**	**ICER (USD/LY)**	**ICER (USD/QALY)**
ITT patients	Nab-paclitaxel	8,524	1.87	1.297	4,558	6,572		
	Atezolizumab + nab-paclitaxel	83,700	2.606	1.724	32,118	48,550	102,141	176,056
PD-L1(+) patients	Nab-paclitaxel	8,007	1.836	1.259	4,361	6,360		
	Atezolizumab + nab-paclitaxel	99,688	3.101	2.035	32,147	48,987	72,475	118,146
PD-L1(–) patients	Nab-paclitaxel	9,211	1.907	1.34	4,830	6,874		
	Atezolizumab + nab-paclitaxel	76,411	2.316	1.548	32,993	49,361	164,303	323,077

*ITT, intention-to-treat; PD-L1, programmed death ligand-1; LY, life-year; QALY, quality-adjusted life-year; CER, average cost-effectiveness ratio; ICER, incremental cost-effectiveness ratio; USD, United States dollar*.

### Scenario 1

In the group of intention-to-treat patients, the base-case analysis results showed that life-year (LY), QALY and cost were 2.606, 1.724, and $83,700 in the atezolizumab plus nab-paclitaxel regimen. 1.87, 1.297, and $8,524 in the nab-paclitaxel regimen. Compared with nab-paclitaxel regimen, patients received atezolizumab plus nab-paclitaxel increased the cost by $75,176 with the augments of 0.736 LYs and 0.427 QALYs. The average cost-effectiveness ratios of atezolizumab plus nab-paclitaxel are $32,118/LY and $48,550/QALY. And that of nab-paclitaxel regimen is $4,558/LYs and $6,572/QALYs. The ICER of atezolizumab plus nab-paclitaxel compared with nab-paclitaxel is $102,141/LY and $176,056/QALY.

### Scenario 2

In the PD-L1 (+) group, the base-case analysis results showed that LY, QALY and cost were 3.101, 2.035, and $99,688 in the atezolizumab plus nab-paclitaxel regimen. 1.836 LY, 1.259 QALYs and $8,007 in the nab-paclitaxel regimen. Patients receiving atezolizumab plus nab-paclitaxel had a $91,681 increase in cost with a rise of 1.265 LYs and 0.776 QALYs compared with nab-paclitaxel alone. The mean cost-effectiveness ratios for atezolizumab plus nab-paclitaxel are $32,147/LY and $48,987/QALY. And nab-paclitaxel regimen is $4,361/LYs and $6,360/QALYs. The ICER for atezolizumab plus nab-paclitaxel vs. nab-paclitaxel is $72,475/LY and $118,146/QALY.

### Scenario 3

In the group of PD-L1(–) patients, LY, QALY and cost were 2.316, 1.548, and $76,411 in the atezolizumab plus nab-paclitaxel regimen. 1.907 LY, 1.34 QALYs and $9,211 in the nab-paclitaxel regimen. In comparison with patients receiving only nab-paclitaxel, atezolizumab plus nab-paclitaxel led to an additional $67,200 in cost with an increase of 0.409 LYs and 0.208 QALYs. The mean cost-effectiveness ratios of atezolizumab plus nab-paclitaxel are $32,993/LY and $49,361/QALY. Nab-paclitaxel regimen is $4,830/LYs and $6,874/QALYs. The ICER for atezolizumab plus nab-paclitaxel vs. nab-paclitaxel is $164,303/LY and $323,077/QALY.

### One-Way Sensitivity Analysis

**Figure 3:**
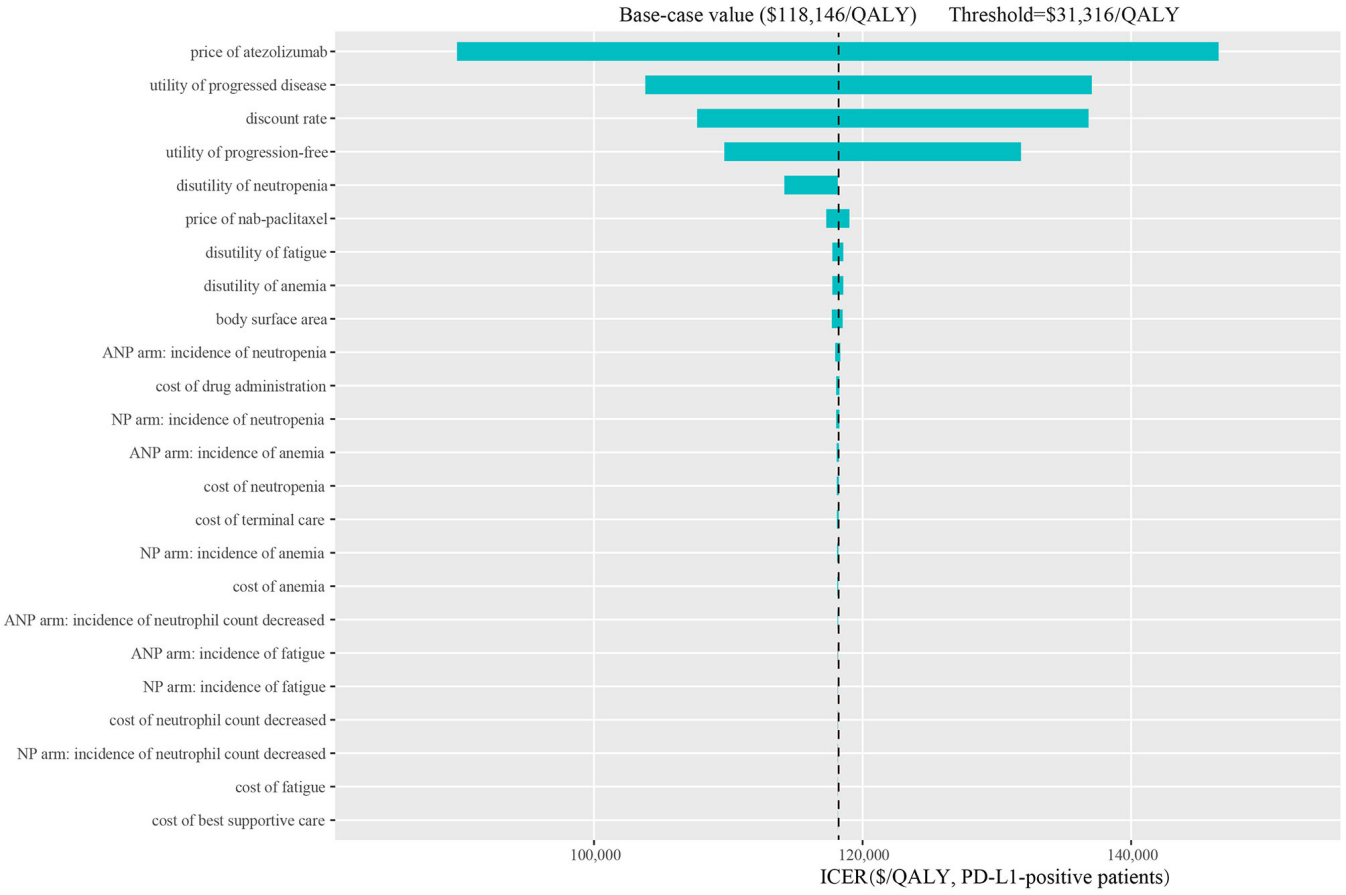
Tornado diagram of one-way sensitivity analysis in the PD-L1-positive patients. ITT, intention-to-treat; PD-L1, programmed death ligand-1; QALY, quality-adjusted life-year; ICER, incremental cost-effectiveness ratio; ANP, atezolizumab plus nab-paclitaxel; NP, nab-paclitaxel.

The one-way sensitivity analyses are shown in the tornado diagrams. In all scenarios, the price of atezolizumab, utility of progressed disease and discount rate have substantial influence on the ICERs between atezolizumab plus nab-paclitaxel and nab-paclitaxel. The range for the one-way sensitivity analysis was from $132,732/QALY to $218,788/QALY in the ITT group (scenario 1, Supplementary Figure 1A). The range of PD-L1-positive group was between $89,792/QALY and $146,514/QALY (scenario 2, Figure 3). PD-L1-negative group ranged between $241,981/QALY and $428,361/QALY (scenario 3, Supplemental Figure 1B).

### Probabilistic Sensitivity Analysis

**Figure 4:**
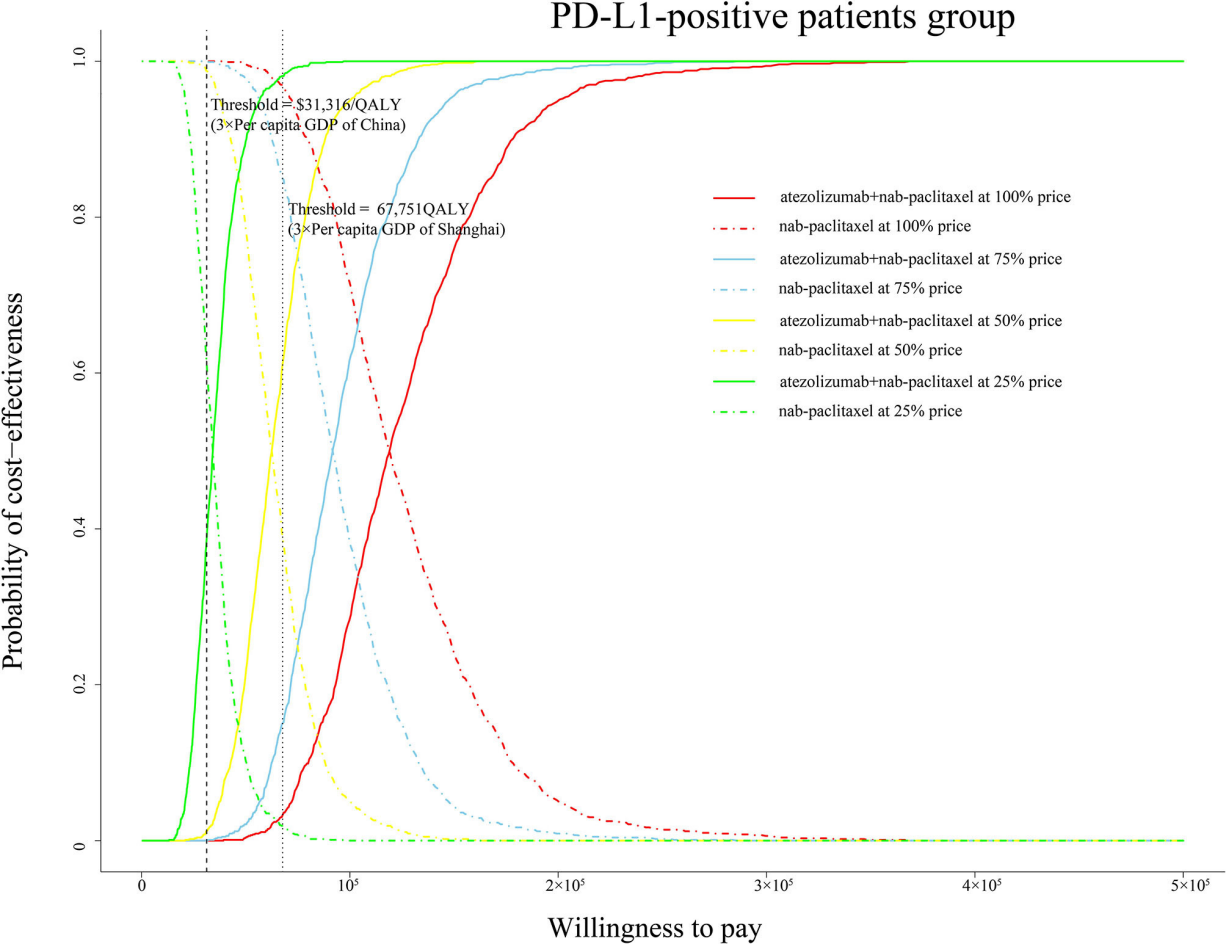
Cost-effectiveness acceptable curve. The y-axis indicates the probability that a regimen is cost-effective across the willingness-to-pay threshold (x-axis). QALY, quality-adjusted life-year; PD-L1, programmed death ligand-1; GDP, gross domestic product.

One thousand iterations were conducted to evaluate all model parameters sampling from probability distributions simultaneously. At the threshold of $31,316/QALY, the cost-effectiveness acceptable curve (CEAC) showed that a 0% likelihood of atezolizumab plus nab-paclitaxel regimen being cost-effective in various groups (ITT, PD-L1-positive and PD-L1-negative patients). In addition, the simulations about adjusting the cost of atezolizumab at 75, 50, and 25% price were also conducted. Notably, the CEAC (Figure 4) indicated that the chance of atezolizumab plus nab-paclitaxel regimen being cost-effective were 0, 1.3, and 38.7% at 75, 50, and 25% price with a WTP threshold of $31,316/QALY in the PD-L1-positive patients. And the CEAC of ITT group (Supplementary Figure 2A) showed 0, 0, and 8.5% likelihood at 75, 50, and 25% price with a WTP threshold of $31,316/QALY. Likewise, nearly 0% probability at 75, 50, and 25% price of atezolizumab in the PD-L1-negative group (Supplementary Figure 2B).

## Discussion

Since TNBC patients express none of these three receptors (ER, PR, and HER2) exhibit a worse prognosis, making anti-tumor immune system re-identify tumor cells through inhibiting the programmed cell death 1 pathway became a solution that can be tried (28). Atezolizumab as a PD-L1 inhibitor was reported to have better clinical benefit for TNBC patients in IMpassion130 trial, which brought hope to both breast cancer patients and clinicians. And then, atezolizumab was approved in February 2020 for the treatment of small cell lung cancer, making it the second PD-L1 inhibitor approved for listing in China. Considering that the indication for triple negative breast cancer has been approved in the United States, approval of treatment for TNBC in China may also be a matter of time.

To explore the economic burden induced by the new treatment regimen, we looked up the bidding price of atezolizumab and performed a cost-effectiveness analysis. To our knowledge, this is the first study to evaluate the health and economic outcomes of treatment with atezolizumab plus nab-paclitaxel vs. paclitaxel regimen in TNBC patients from the perspective of Chinese health sector. Our base-case analysis showed that the ICER of atezolizumab plus nab-paclitaxel vs. nab-paclitaxel in the ITT, PD-L1(+) and PD-L1(–) group is, respectively, $176,056/QALY, $118,146/QALY, and $323,077/QALY. The ICER values exceed the average threshold of $31,316/QALY in China. The most obvious finding to emerge from the analysis result is that the PD-L1-positive group gets better advantage of survival. One-way sensitivity analysis showed the range of ICER was from $89,792/QALY to $146,514/QALY in the PD-L1-positive group. The lowest value still exceeds the threshold of $31,316/QALY. Tornado diagram of PD-L1-positive group demonstrated that the price of atezolizumab was the most influential model input. Utility of PD, discount rate and utility of PF were also found to be key factors. Our probabilistic sensitivity analysis additionally simulated various conditions covered the price of atezolizumab reduced by 25, 50, and 75%. If adjust the price of atezolizumab to 25% in the PD-L1-positive group, the likelihood of atezolizumab plus nab-paclitaxel regimen being cost-effective is 38.7%. In the case of the current threshold, the price needs to be reduced by a large margin. If the threshold of $67,751/QALY in Shanghai is used as a comparative standard, then the probability of cost-effectiveness is, respectively, 15.1, 61, and 98.1% at the 75, 50, and 25% of price of atezolizumab. The results reflect that the price of atezolizumab is too high and the average WTP threshold in China is low, which makes it difficult to change the conclusion that atezolizumab plus nab-paclitaxel regimen is not cost-effective choice.

Currently, we know that some researcher conducted economic evaluations from the point of view of other countries. Wu et al. found that atezolizumab is cost-effective option for PD-L1-positive patients with a threshold of $200,000/QALY in the US (29). However, another US-based study conducted by Li et al. showed that atezolizumab plus nab-paclitaxel were not considered cost-effective regimen (30). Phua et al. concluded that adding atezolizumab to nab-paclitaxel was not cost-effective for treatment of PD-L1-positive TNBC based on the context of Singapore (31). Although the cost-effectiveness of atezolizumab plus chemotherapy based on nab-paclitaxel regimen differs from various views, these evaluations all implied that the price of atezolizumab is too high. With the demand for treatment of TNBC soaring, there is still room for price reduction in the future.

Our study has limitations. First, our survival data derived from IMpassion130, in which the patients were predominantly white. Asian patients accounted for a relatively low proportion. However, our study was made from the perspective of China. Inevitably, the analysis result was slightly affected by race. Second, our study was conducted based on modeling techniques. IPD applied to model was not actual IPD but projected IPD generated according to the specific algorithm. Then, analysis results using parametric model to extrapolate the survival outcomes beyond the time horizon could have a slight hypothesis bias compared with analysis results with sufficient survival data of follow-up. It could undermine the robustness, but our sensitivity analyses covered substantial ranges of all variables. It can well foresee some changes in results induced by modeling techniques. Finally, no research about quality-of-life was conducted in IMpassion130 trial, direct data sources were lacked. Therefore, the utility data were determined based on published literatures. It would lead to deviations in the cumulative QALYs and may be distinct from actual quality- of-life data. In addition to limitations, we made many efforts in the stage of selecting models. We considered the Markov model, partitioned survival model and the cure model (32). According to the characteristics of the survival curve, we exclude the application of the cure model. Likewise, in order to reduce the deviation caused by the hypothesis, we finally chose the latter between the Markov model and the partitioned survival model. The partitioned survival model could directly obtain survival cohort proportion from survival curve, which can decrease the hypothesis bias of calculating transition probability of PF or PD state to death state.

## Conclusions

In summary, the addition of atezolizumab to nab-paclitaxel could improve the survival time significantly in the PD-L1-positive group, but it is not a cost-effective strategy compared to nab-paclitaxel monotherapy for patients with advanced or metastatic triple-negative breast cancer in the current economic context of China.

## Data Availability Statement

The original contributions presented in the study are included in the article/Supplementary Material, further inquiries can be directed to the corresponding author/s.

## Ethics Statement

This study was based on a literature review and modeling techniques. This study didn't require approval by an Institutional Research Ethics Board.

## Author Contributions

XL and YZ was involved in the design of the study. YLa collected the data, performed the economic analysis, and wrote the first draft of the manuscript. YLi collected data and verified results. All authors approved this version for publication.

## Conflict of Interest

The authors declare that the research was conducted in the absence of any commercial or financial relationships that could be construed as a potential conflict of interest.

## Publisher's Note

All claims expressed in this article are solely those of the authors and do not necessarily represent those of their affiliated organizations, or those of the publisher, the editors and the reviewers. Any product that may be evaluated in this article, or claim that may be made by its manufacturer, is not guaranteed or endorsed by the publisher.
