# Supplementary material for: Atezolizumab Plus Chemotherapy vs. Chemotherapy in Advanced or Metastatic Triple-Negative Breast Cancer: A Cost-Effectiveness Analysis
Source: Front Public Health. 2021 Oct 29;9:756899. doi: 10.3389/fpubh.2021.756899 (PMC8585931; doi:10.3389/fpubh.2021.756899)
Supplement: Supplementary file 1 [file Data_Sheet_1.docx]

Supplemental figure 1A. Tornado diagram of one-way sensitivity analysis in ITT patients.

ITT: intention-to-treat; QALY: quality-adjusted life-year; ICER: incremental cost-effectiveness ratio; ANP: atezolizumab plus nab-paclitaxel; NP: nab-paclitaxel.

Supplemental figure 1B. Tornado diagram of one-way sensitivity analysis in PD-L1-negative patients.

ITT: intention-to-treat; PD-L1: programmed death ligand-1; QALY: quality-adjusted life-year; ICER: incremental cost-effectiveness ratio; ANP: atezolizumab plus nab-paclitaxel; NP: nab-paclitaxel.

Supplemental figure 2A. cost-effectiveness acceptable curve. The y-axis indicates the probability that a regimen is cost-effective across the willingness-to-pay threshold (x-axis).

QALY: quality-adjusted life-year; ITT: intention-to-treat; GDP: gross domestic product.

 Supplemental figure 2B. cost-effectiveness acceptable curve. The y-axis indicates the probability that a regimen is cost-effective across the willingness-to-pay threshold (x-axis).

QALY: quality-adjusted life-year; PD-L1: programmed death ligand-1; GDP: gross domestic product.
